# Tumour Versus Germline *BRCA* Testing in Ovarian Cancer: A Single-Site Institution Experience in the United Kingdom

**DOI:** 10.3390/diagnostics11030547

**Published:** 2021-03-19

**Authors:** Iolia Akaev, Siavash Rahimi, Olubukola Onifade, Francis John Edward Gardner, David Castells-Rufas, Eleanor Jones, Shyamika Acharige, Chit Cheng Yeoh

**Affiliations:** 1 School of Pharmacy and Biomedical Sciences, University of Portsmouth, St. Michaels Building, White Swan Road, Portsmouth PO1 2DT, UK; rahimi.siavash@gmail.com; 2 Department of Histopathology, Brighton and Sussex University Hospitals NHS Trust, Royal Sussex County Hospital, Brighton BN2 5BE, UK; 3 Department of Cellular Pathology, Portsmouth Hospitals University NHS Trust, Southwick Hill Road, Portsmouth PO6 3LY, UK; olubookey@yahoo.com; 4 Department of Obstetrics and Gynaecology, Portsmouth Hospitals University NHS Trust, Southwick Hill Road, Portsmouth PO6 3LY, UK; Gardner.gynaecology@gmail.com; 5 Microelectronics and Electronic Systems Department, Universitat Autònoma de Barcelona, Carrer de les Sitges s/n, 08193 Bellaterra, Spain; David.Castells@uab.cat; 6 Department of Oncology, Portsmouth Hospitals University NHS Trust, Southwick Hill Road, Portsmouth PO6 3LY, UK; Eleanor.Jones@porthosp.nhs.uk (E.J.); Shyamika.Acharige@porthosp.nhs.uk (S.A.); ChitCheng.Yeoh@porthosp.nhs.uk (C.C.Y.)

**Keywords:** *BRCA1*, *BRCA2*, tumour, somatic, carcinoma, ovarian, service, NGS, PARP

## Abstract

The aim of this audit was to evaluate the usefulness and serviceability of testing for pathogenic mutations in *BRCA1* or *BRCA2* (*BRCA1/2*) genes in ovarian cancer (OC) patients. One hundred and thirty-five patients with more common histological sub-types of OC were retrospectively identified between 2011 and 2019. The fail rate of the molecular analysis was 7.4% (10/135). One hundred and twenty-five records were evaluated: 99 (79.2%) patients had wild-type *BRCA* (both somatic and germline); tumour *BRCA1/2* (t*BRCA1/2*) pathogenic mutations were found in 20 (16%) patients with distribution between *BRCA1* and *BRCA2* being 40% and 60%, respectively; 13 (10.4%) patients with pathogenic variants had germline mutations; and t*BRCA1/2* with variant of unknown significance (VUS), in the absence of pathogenic *BRCA1* or *BRCA2* variants, was detected in 6 (4.8%) patients. Our data show that expanding the molecular service to the routine first-tumour testing for patients with OC will potentially increase the detection rate of *BRCA* mutations, thereby providing early benefits of PARP inhibitors therapy. The tumour testing service should continue to be offered to newly diagnosed patients with high-grade epithelial cancers, including high-grade serous carcinoma, but also with carcinosarcomas and poorly-differentiated metastatic adenocarcinomas of unknown origin.

## 1. Introduction

Ovarian carcinoma (OC) is a significant cause of mortality in women. In 2020, there were an estimated 8267 new cases of OC in the United Kingdom (UK), with the mortality rate of 4063 [1]. In the USA, approximately 22,500 women are diagnosed each year, with a mortality rate of 62.2% [2].

*BRCA1* and *BRCA2* (*BRCA1/2*) are tumour suppressor genes, involved in the homologous repair of double-stranded DNA breaks, located at 17q21.31 and 13q13.1, respectively [3,4]. Approximately 75% of women with hereditary breast and ovarian cancers have germline *BRCA1* mutations (g*BRCA1*), and 10–20% have germline *BRCA2* (g*BRCA2*) mutations [5]. The lifetime risk of developing OC in women with germline *BRCA* (g*BRCA*) mutations is estimated at 40–60%. 

In the general population, approximately 15% of OC cases are associated with g*BRCA1* or g*BRCA2* (g*BRCA1/2*) pathogenic variants (PVs), and additional 5% show somatic *BRCA* (s*BRCA*) mutations in one of these genes [6,7]. Tumour *BRCA1/2* (t*BRCA1/2*) tissue analysis can detect both s*BRCA* and g*BRCA* pathogenic mutations [8,9].

In the last few years, different studies have reported on their experience in the implementation of t*BRCA1/2* testing into clinical-diagnostic routine practice [10,11,12,13,14]. The overall experience appears positive, and results are promising for the implementation of the first-tumour testing approach in contrast to the standard model of germline-first testing, although some recommendations were published in regard to the paired tumor-normal (blood) parallel testing [8]. Paired sequencing can differentiate somatic and germline variants, but its implementation into standard practice can be challenging. This is mainly due to the expense of molecular testing of two samples per patient, limited tumour material, difficulties in organising pathology services to perform tumor testing, or acquiring both tumour and blood sample in a reasonable period of time. However, paired tumor-normal analysis is the only way to distinguish somatic from inherited germline mutations in results from tumour testing [9].

It is important to know the *BRCA* status, because mutations in *BRCA1/2* confer enhanced sensitivity to treatment with poly (ADP-ribose) polymerase (PARP) inhibitors (PARPi) [15]. Currently, PARPi are typically used in the treatment of OC, either as maintenance therapy or as single-agent therapy for recurrent disease, following the response to the initial course of platinum-based therapy in patients with g*BRCA1/2*- or s*BRCA1/2*-mutated OC. Many drugs have been licensed for this purpose, including Olaparib (Lynparza^®^), Niraparib (Zejula^®^) and Rucaparib (Rubraca^®^).

In this work, we set the objectives to evaluate the prevalence of pathogenic *BRCA1/2* germline and somatic mutations patients with ovarian cancer in population of Portsmouth, UK, and to evaluate the usefulness and serviceability of t*BRCA1/2* mutations analysis. Moreover, we aim to evaluate parallel, first-tumour or germline-first analysis pathways. 

## 2. Materials and Methods

Patients diagnosed with OC were retrospectively identified by oncologists through the audit of their clinical database between 2011 and 2019 (Audit ID: 4975; “Evaluation of the serviceability of a concomitant/parallel germline and tumour testing for *BRCA1/2* variants for patients with ovarian cancer’’). The cohort included patients diagnosed with high-grade serous carcinoma (HGSC), endometrioid adenocarcinoma (EAdCa), clear cell carcinoma (CCC), carcinosarcoma and poorly-differentiated metastatic adenocarcinoma of unknown origin (Met. AdCa (G3)). All patients had already been tested for tumour and germline mutations in *BRCA1/2* as part of the diagnostic service provision.

The g*BRCA1/2* testing was performed on the blood samples. The service was provided by regional Medical Genetics Service Molecular Laboratory using the next-generation sequencing (NGS) of all coding sequences and exon/intron boundaries of *BRCA1* and *BRCA2* on Illumina MiSeq.

The tumour testing service was provided by AstraZeneca at the contracted Genomics Diagnostic Laboratories, as part of the tumour *BRCA1/2* screening on OCs for trusts, governed by the National Health Service (NHS) England. Testing was carried out on formalin-fixed paraffin-embedded (FFPE) tissue, based on the availability of surplus diagnostic material. Tumour testing was performed utilising the NGS analysis of the coding regions of *BRCA1* and *BRCA2* (OMIM Number(s) 604370 and 612555, respectively) using multiplex PCR-based target enrichment GeneRead DNAseqv2 Human Breast Cancer Panel (Qiagen, Hilden, Germany); constructed NGS libraries were sequenced on Illumina HiSeq. Mutation and variant calling was performed by custom bioinformatic analysis pipeline, validated to detect Single Nucleotide Variants and small insertion/deletion mutations (<40 bp) to 5% mutant allele frequency (MAF). Mutations were named according to Human Genome Variation Society guidelines (https://varnomen.hgvs.org/, accessed on 31 January 2020), using reference sequences LRG_292 and LRG_293.

## 3. Results

Results of tumour testing were available for 135 patients, out of which 10 had failed analysis (7.4%). Samples with failed analysis were mainly paraffin blocks from surgical resection specimens (*n* = 6), followed by biopsies (*n* = 3) and a cell clot from ascites (*n* = 1). Tumour cellularity was mainly 50–80% (*n* = 7) and 20–30% (*n* = 3). The time in archives was also variable between 3 and 6 years (*n* = 8), 8 years (*n* = 1) and 1 year (*n* = 1). Three out of the ten failed cases reported presence of pathogenic mutations in the germline. 

The final number of patients who had result from the tumour testing was 125: 94 had HGSC (75.2%), 17 (13.6%) had EAdCa, seven (5.6%) had CCC, four (3.2%) had carcinosarcoma and three (2.4%) had Met. AdCa (G3) (Table 1). 

Ninety-nine cases were wild-type *BRCA1/2* (both somatic and germline), representing 79.2% of all tested cases. t*BRCA1/2* with variant of unknown significance (VUS), in the absence of pathogenic *BRCA1* or *BRCA2* variants, was found in six cases (4.8%), out of which three cases had the same VUS detected in the germline. Thirteen cases (10.4%) showed both g*BRCA1/2* and s*BRCA1/2* PVs, out of which six cases (46%) were *BRCA1*-mutated and seven cases (54%) were *BRCA2*-mutated. Seven cases (5.6%) showed wild-type g*BRCA1/2* and pathogenic s*BRCA1/2*.

In total, t*BRCA1/2* PVs were found in twenty cases (16%) with distribution between *BRCA1* and *BRCA2* being 40% and 60%, respectively. 

The positive cases were divided into three subgroups, as shown in Table 2, and are as follows:

- Subgroup 1: six patients with VUS in *BRCA1/2*.
- Subgroup 2: thirteen patients with g*BRCA1/2* PVs and s*BRCA1/2* PVs.
- Subgroup 3: seven patients with wild-type g*BRCA1/2* and s*BRCA1/2* PVs.

The majority of the pathogenic mutations reported were single nucleotide variants with frameshift, missense or nonsense molecular consequences (Table 2).

The mean age was 64 years (range 31–92 years). The mean age of patients with g*BRCA* PVs was 63 years (range 51–76 years), and with s*BRCA* PVs it was 61 years (range 49–70 years). The mean age of patients with wild-type *BRCA* was 65 years (range 31–92 years).

## 4. Discussion

Patients with advanced OC have a poor prognosis, with a 5-year overall survival rate of 45% [1], and recurrence within the first 6 months following chemotherapy expected in 20–30% of cases [16]. It is generally accepted that patients with *BRCA*-mutated OCs are more likely to respond to platinum-based chemotherapy and benefit from PARPi therapy [15]. Three randomised phase III trials (NOVA, ARIEL3 and SOLO1) confirmed that PARP inhibition is a highly effective maintenance treatment for patients with platinum-sensitive recurrent OC, with the greatest benefit observed in patients with *BRCA*-mutated OCs who demonstrated higher survival rate compared to patients with sporadic OCs. All three trials also observed anti-tumour activity in patients with measurable disease at the initiation of maintenance therapy, suggesting the indication for PARP inhibition as initial therapy, as well as maintenance therapy [17,18,19].

Moreover, the matured data of SOLO1 has demonstrated that in OC patients with g*BRCA* mutations, and complete or partial response to platinum chemotherapy, the risk of disease progression or death was lower by 70% among the Olaparib group, compared with the placebo group [19]. This has led to a recommendation by the UK National Institute for Health and Care Excellence (NICE) for all patients with *BRCA*-mutated OCs to be eligible for Olaparib maintenance therapy [20]. Thereafter, it was advised to refer all OC patients for g*BRCA* testing prior to initial therapy, in order to determine their suitability for PARPi/maintenance therapy. First-line maintenance treatment with PARPi is available through the Cancer Drugs Fund for people with a *BRCA1* or *BRCA2* gene mutation. However, there is no first-line PARPi/maintenance therapy for people without a pathogenic mutation in *BRCA1* or *BRCA2* genes. As a result, the routine surveillance is the only option for these patients.

Recently, Niraparib has been recommended for use within the Cancer Drugs Fund as an option for maintenance treatment for advanced (FIGO stages 3 and 4) high-grade epithelial ovarian, fallopian tube or primary peritoneal cancer after response to first-line platinum-based chemotherapy in adults [21]. The recommendation was provided following the results of PRIMA study [22]. Niraparib will be the first PARPi/maintenance therapy that will be offered to ovarian cancer patients with advanced disease, regardless of *BRCA* mutation status. Therefore, in our view, following the recent advancement in the availability of maintenance options, it is important to know the tumour and germline status, as it will allow to stream patients into correct maintenance options from the start of their treatment journey. 

The rate of g*BRCA* PVs has been reported in the range of 8–34% in different studies. The Cancer Genome Atlas Research Network found mutations in 22% of a series of 489 cases [23], while Gornjec et al. found mutations in 34% of a relatively small series of 44 patients [24]. In our series, the rate of germline mutations was 10.4%, which is comparable to the figure of 7.8% reported by Plaskocinska et al. in an East Anglia region study of 232 cases [25]. Additionally, Rahman et el. reported 14.3% in a London region (UK) study of 122 cases [26], George et al. reported 16% in The Royal Marsden (UK) study [27] and 14.1% was reported by Alsop et al. in an Australian study of 1001 cases [6].

The rate of t*BRCA* PVs in HGSC was 21.3% in the report by McCuaig et al. in the Canadian study [14]; however, the authors have not specified the rate of germline mutations in those identified tumours, therefore the somatic prevalence cannot be established. In our group of epithelial OCs, the rate of t*BRCA* PVs was 16%, which is similar to 16.7% reported by Vos et al. in the Netherlands study of t*BRCA1/2* testing of 315 patients [10] and comparable with 17.4% reported by Turashvili et al. in the Canadian study of 276 patients [12].

The rate of s*BRCA* mutation in HGSC was 2% and 3% in the reports of Cancer Genome Atlas Research Network [23] and Rahman et al. [26], respectively. Gornjec et al. found 4.5% [24]. Geisler et al. found s*BRCA* mutations in 6.8% in all types of OCs [28]. We found prevalence of s*BRCA* mutations in 5.6% of 5 HGSC and 2 EAdCa patients with a wild-type g*BRCA*. The s*BRCA* mutations in these women would have remained undetected with germline test only. Although this rate is relatively low, it has significant therapeutic benefits. 

The possible reason for the lower rate of g*BRCA* and s*BRCA* mutations in our results with respect to other studies [14,24,27,28] might be that we were limited to the population of Portsmouth, UK. Possibly, in this population, the *BRCA1/2* mutations are less prevalent, compared to the populations described in other studies. 

Alsop et al. did not find *BRCA1/2* mutations in their series of thirty-four patients with carcinosarcoma [6]. Rahman et al. found one carcinosarcoma patient with germline mutation [26]. We have found one carcinosarcoma (epithelial component had morphology of serous carcinoma and sarcomatous component included chondrosarcomatous heterologus elements) with pathogenic mutations (t*BRCA* PVs were confirmed by g*BRCA*), and another carcinosarcoma (epithelial component had morphology of serous carcinoma) with VUS in *BRCA1* gene. Therefore, based on our experience with a small number of cases, *BRCA* testing should be considered in ovarian carcinosarcomas, especially in those cases where the epithelial component is of serous morphology.

Reversion of mutated *BRCA1* and *BRCA2* to the wild-type genes has been reported [29,30] and may be associated with acquired resistance to PARPi. We have evaluated available results of paired tumour samples in five patients with HGSC, three with wild-type *BRCA* and two with a g*BRCA1* mutation, and have not encountered changes in *BRCA* mutation status between tumour samples taken pre- and post-clinical relapse (four patients had PARPi/maintenance therapy and only one chemotherapy prior to clinical relapse); however, the number of patients is too low to allow conclusions to be drawn. Nevertheless, the number of detected allele frequency reads of *BRCA1* mutation following clinical relapse was higher, compared to its baseline: 93% vs. 70% for one patient and 68% vs. 40% for the second patient. This is further proof that detected pathogenic *BRCA1* is a driver mutation in these tumours. Oncologists should consider tumour testing in relapsed specimens.

Tumour tissue testing can potentially identify somatic or germline mutations, or both somatic and germline [8,9]. Germline mutations must be confirmed by a subsequent blood testing, because detected germline mutations can have consequences for all family members of the tested patient. The sole s*BRCA* mutation does not generally present any implications for the patient’s family, as only the tumour cells have *BRCA* mutations [31].

Consenting for germline testing requires additional training for oncologists and a referral to genetic counselling, whereas it is not required for somatic testing. Informed consent is required for germline testing, whereas a verbal discussion is sufficient for the tumour testing (documenting discussion in the notes), followed by the use of the standard Trust consent form for the biopsy/surgery, which covers testing on samples, rather than a separate consent form (G Crawford 2021, personal communication, 29 January 2021). Patients with detected PVs are usually referred to genetic counselling, whereas patients with identified VUS do not always get a referral, commonly due to the difficulties in interpretation of VUS in relation to clinical decisions. It is recommended that oncologists should refer all cases with identified VUS to the clinical genetic services as it will provide clinically relevant classification for each reported *BRCA* variant and inform the patient [31].

Germline test results can be expected after six to eight weeks, in contrast to somatic results, which can be reported within two to four weeks, provided the tumour sample contains sufficient DNA for analysis. It is worth mentioning that testing tumour from the FFPE tissue blocks may potentially provide inconclusive results mainly due to technical reasons, such as poor quality or insufficient tumour cell DNA in the analysed tumour samples due to long period of storage [32]. In our audited series, analytical failure rate was 7.4%, which was mainly due to low DNA yields and highly fragmented DNA, most likely caused by formalin fixation and storage of FFPE blocks.

In our audit, the rate of detection of wild-type g*BRCA* was relatively high (99 out of 125 patients). Therefore, initial tumour testing potentially would make the germline testing unnecessary in approximately 79% of patients. It was reported by Vos et al. that initial tumour testing approach for all newly diagnosed patients in the Netherlands found a 38.7% reduction in the number of required genetic tests and concomitant genetic counselling, as compared to germline-first models [10].

Interestingly, D’Indinosante et al. tested the approach of initial tumour testing, followed by the germline testing, to identify whether some cases could have been missed. Three hundred and five patients were tested, and t*BRCA* was identified in 106 (35%). Concomitantly, g*BRCA* PVs was confirmed in 89 (88%) patients and showed a wild-type variant in 12 patients (12%). Pathogenic germline mutations were not detected in all wild-type t*BRCA* cases that were tested [11].

Therefore, the initial tumour testing can be implemented without concerns of missing patients with germline *BRCA* pathogenic mutations. In our audit, all tumours with wild-type *BRCA* were also germline wild-type.

In contrast, in the consensus paper published by British Association of Gynaecological Pathologists (BAGP), it was suggested that parallel tumour and germline testing should be implemented. This is due to a concern that pathogenic large genomic rearrangements (LGRs), which can be detected in germline samples using NGS, can be missed when using FFPE-derived tumour DNA samples, because of a high analytical failure rate. It was reported that 10–15% of patients with g*BRCA* PVs were not detected in the tumour testing [8]. In our audit, in the failed analysis group 3% of patients had g*BRCA* PVs. However, all failed samples in our audit had germline testing. Therefore, it is reasonable to say that the first-tumour testing with subsequent germline testing of patients, harbouring t*BRCA* mutations (pathogenic and VUS), or where testing of tumour samples has failed for technical reasons, should be the initial approach over the germline-first model for selecting patients for PARPi/maintenance therapy. However, in the event, where no variant has been identified in the tumour testing, a blood test still can be initiated (with consideration for the family history) for the definitive confirmation of absence of any variants. This approach would save the cost of the associated genetic counseling and reduce the number of required genetic tests.

We believe that the most frequent reason for selecting germline testing over tumour testing is resource implications due to additional workload for pathology laboratories. Tumour testing was provided by AstraZeneca via the BRCAct service [33] and was performed at a service laboratory (Manchester Genomics), which accepted material in the form of slides or paraffin blocks. Preparation of slides necessitates additional work for the local laboratory, such as selection, retrieval and sectioning of the blocks with adherence to molecular good practice protocols, which may be an issue in a busy service-orientated laboratory, unless it has a dedicated cutting station for specimens requiring molecular tests. We have found that approximately three to four hours of preparation time are required for two to three cases per week. Some staff time can be saved if paraffin blocks are sent instead of slides, but in that case there is risk of delay if the block is required for another purpose, such as additional testing or clinical trials, or it can be lost in transit, with subsequent delay in a potential PARPi/maintenance therapy for patients.

Nevertheless, we have established from the results that the tumour testing service is useful, as it can identify more patients suitable for PARPi/maintenance therapy and it can help all patients with advanced epithelial ovarian cancers with confirmed *BRCA* status (either with pathogenic mutations or wild-type) to enter the appropriate treatment pathway at the time of initial diagnosis. Tumour testing can be serviceable by local pathology laboratories if the tumour testing will be provided by contracted Genomics Diagnostic Laboratories. In the era of molecular pathology, the first-tumour testing should be incorporated into the diagnostic service pathway. Additionally, the first-tumour testing pathway would potentially help the oncology and genetic services to decrease the time associated with consenting, genetic counselling and the number of genetic tests.

## 5. Conclusions

In summary, we have demonstrated that expanding the service to include routine tumour testing for patients with OC will increase the detection rate of *BRCA* mutations, thereby providing benefits of available PARPi/maintenance therapy. The main limitation of this report was the sample size. The tumour testing service should continue to be offered to newly diagnosed patients with high-grade epithelial cancers, including HGSC, but also with carcinosarcomas and poorly-differentiated metastatic adenocarcinoma of unknown origin.

## Figures and Tables

**Table 1:** The distribution of results between different tumour subtypes from *BRCA1/2* testing.

Results	HGSC ^1^	EAdCa ^2^	CCC ^3^	CRS ^4^	Met. AdCa (G3) ^5^	Total
WT ^6^ g*BRCA* and s*BRCA*	77	13	5	2	2	99 (79.2%)
WT g*BRCA* and VUS ^7^ in s*BRCA*	0	1	2	0	0	3 (2.4%)
VUS in g*BRCA* and s*BRCA*	1	1	0	1	0	3 (2.4%)
Pathogenic g*BRCA*m and s*BRCA*m	11	0	0	1	1	13 (10.4%)
WT g*BRCA* and pathogenic s*BRCA*m	5	2	0	0	0	7 (5.6%)
Total	94 75.2%	17 13.6%	7 5.6%	4 3.2%	3 2.4%	125 100%

^1^ HGSC = high-grade serous carcinoma; ^2^ EAdCa = endometrioid adenocarcinoma; ^3^ CCC = clear cell carcinoma; ^4^ CRS = carcinosarcoma; ^5^ Met. AdCa (G3) = metastatic poorly differentiated adenocarcinoma; ^6^ WT = wild-type; ^7^ VUS = variants of unknown significance.

**Table 2:** Results from patients with detected variants of unknown significance (VUS) (samples 1–6), and pathogenic variants in g*BRCA1/2* and s*BRCA1/2* (samples 7–19), or s*BRCA1/2* (samples 20–26).

№	Diagnosis	Tissue	g*BRCA1/2* Status	s*BRCA1/2*: VUS ^1^ or Pathogenic Mutations
1	CCC ^2^	Adnexum *	WT ^3^	Not detected, but VUS in 32% of reads in *BRCA1* c.4186-11 C>T; not identified in germline.
2	HGSC ^4^	Adnexum	VUS	Not detected, but VUS in *BRCA1* c.1616C>T p.(Thr539Met); same VUS identified in germline.
3	EAdCa ^5^	Ovary	VUS	Not detected, but VUS in *BRCA2* c.1216G>A p(Ala406Thr), c1219C>G p(Gln407Glu); same VUS identified in germline.
4	EAdCa	Ovary	WT	Not detected, but VUS in *BRCA1* c.4778>C p.(Ile1593Thr); not identified in germline.
5	CRS ^6^	Adnexum	VUS	Not detected, but VUS 58% of reads in *BRCA1* c.1598A>G p.(Asn533Ser); same VUS identified in germline.
6	CCC	Ovary	WT	Not detected, but VUS in 63% of reads in *BRCA2* c.8350C>T p.(Arg2784Trp); not identified in germline.
7	HGSC	Ovary	*BRCA1*m ^7^	40% reads, *BRCA1* pathogenic frameshift mutation c.4791delC p.(Ser1598LeufsTer3)
8	HGSC	Adnexum	*BRCA2*m ^8^	77% reads, *BRCA2* pathogenic frameshift mutation c.5303_530delTT p.(Leu1768ArgfsTer5)
9	HGSC	Ovary	*BRCA1*m	91% reads, *BRCA1* pathogenic missense mutation c.4868C> p.(Ala1623Gly)
10	HGSC	Ovary	*BRCA1*m	39% reads, *BRCA1* pathogenic frameshift mutation c.1823_1826delAGAA p.(Lys608IlefsTer3)
11	HGSC	Omentum	*BRCA2*m	57% reads, *BRCA2* pathogenic nonsense mutation c.2409T>G p.(Tyr803Ter)
12	Met.AdCa (G3) ^9^	Omentum	*BRCA2*m	58% reads, *BRCA2* pathogenic nonsense mutation c.9294C>G p.(Tyr3098Ter)
13	HGSC	Omentum	*BRCA2*m	89% reads, *BRCA2* pathogenic missense mutation c.7988A>T p.(Glu2663Val)
14	HGSC	Omentum	*BRCA2*m	91% reads, *BRCA2* pathogenic missense mutation c.8167G>C p.(Asp2723His)
15	CRS	Adnexum	*BRCA1*m	91% reads, *BRCA1* pathogenic missense mutation c.4868C>G p.(Alal623Gly)
16	HGSC	Ovary	*BRCA1*m	70% reads, *BRCA1* pathogenic missense mutation c.5095C>T p.(Argl699Trp)
17	HGSC	Cell clot **	*BRCA2*m	22% reads, *BRCA2* pathogenic mutation c.6468_6469deITC p.(GIn2157llefsTer18)
18	HGSC	Ovary	*BRCA1*m	66% reads, *BRCA1* pathogenic missense mutation c.4964C>T p.(Ser1655Phe)
19	HGSC	Omentum	*BRCA2*m	59% reads, *BRCA2* nonsense mutation c.5682C>G p.(Tyr1894Ter)
20	HGSC	Ovary	WT	45% reads, *BRCA2* pathogenic splicing mutation c.8632+1 G>T
21	HGSC	Ovary	WT	64% reads, *BRCA1* pathogenic frameshift mutation c.5147_5150delATTT p.(Tyr1716SerfsTer3)
22	HGSC	Omentum	WT	52% reads, *BRCA2* pathogenic frameshift mutation c.6010_6019del p.(GIu2004ProfsTer33)
23	HGSC	Ovary	WT	73% reads, *BRCA1* nonsense mutation c.1687C>T p.(Gln563Ter)
24	HGSC	Omentum	WT	44% reads, *BRCA2* frameshift mutation c.656_662del p.(Thr219IlefsTerr9)
25	EAdCa	Omentum	WT	42% reads, *BRCA2* nonsense mutation c.5404C>T p.(Gln1802Ter)
26	EAdCa	Ovary	WT	8% reads, BRCA2 nonsense mutation c.6385G>T p.(Glu2129Ter)

^1^ VUS = variants of unknown significance; ^2^ CCC = clear cell carcinoma; ^3^ WT = wild-type; ^4^ HGSC = high-grade serous carcinoma; ^5^ EAdCa = endometrioid adenocarcinoma; ^6^ CRS = carcinosarcoma; ^7^ *BRCA1*m/^8^ *BRCA2*m = pathogenic mutation in *BRCA1* or *BRCA2* genes; ^9^ Met. AdCa (G3) = metastatic poorly differentiated adenocarcinoma; * = the term ‘’adnexum’’ is used because the primary site (ovary or fallopian tube) cannot be established; ** = cell clot from ascites.

## Data Availability

All Data has been included and presented in this article.
